# Isolation and Chimerization of a Highly Neutralizing Antibody Conferring Passive Protection against Lethal *Bacillus anthracis* Infection

**DOI:** 10.1371/journal.pone.0006351

**Published:** 2009-07-24

**Authors:** Ronit Rosenfeld, Hadar Marcus, Einat Ben-Arie, Bat-El Lachmi, Adva Mechaly, Shaul Reuveny, Orit Gat, Ohad Mazor, Arie Ordentlich

**Affiliations:** 1 Departments of Biochemistry and Molecular Genetics, Israel Institute for Biological Research, Ness-Ziona, Israel; 2 Department of Biotechnology, Israel Institute for Biological Research, Ness-Ziona, Israel; 3 Department of Infectious Diseases, Israel Institute for Biological Research, Ness-Ziona, Israel; Technical University Munich, Germany

## Abstract

Several studies have demonstrated that the passive transfer of protective antigen (PA)-neutralizing antibodies can protect animals against *Bacillus anthracis* infection. The standard protocol for the isolation of PA-neutralizing monoclonal antibodies is based upon a primary selection of the highest PA-binders by ELISA, and usually yields only few candidates antibodies. We demonstrated that by applying a PA-neutralization functionality-based screen as the primary criterion for positive clones, it was possible to isolate more than 100 PA-neutralizing antibodies, some of which exhibited no measurable anti-PA titers in ELISA. Among the large panel of neutralizing antibodies identified, mAb 29 demonstrated the most potent activity, and was therefore chimerized. The variable region genes of the mAb 29 were fused to human constant region genes, to form the chimeric 29 antibody (cAb 29). Guinea pigs were fully protected against infection by 40LD_50_ *B. anthracis* spores following two separate administrations with 10 mg/kg of cAb 29: the first administration was given before the challenge, and a second dose was administered on day 4 following exposure. Moreover, animals that survived the challenge and developed endogenous PA-neutralizing antibodies with neutralizing titers above 100 were fully protected against repeat challenges with 40LD_50_ of *B. anthracis* spores. The data presented here emphasize the importance of toxin neutralization-based screens for the efficient isolation of protective antibodies that were probably overlooked in the standard screening protocol. The protective activity of the chimeric cAb 29 demonstrated in this study suggest that it may serve as an effective immunotherapeutic agent against anthrax.

## Introduction

The anthrax bio-terror incidents in the USA clearly demonstrated the threat posed by the intentional release of infectious agents, and highlighted the need for improved prophylactic approaches against anthrax [1], [2]. In a post-exposure scenario, the efficacy of the current FDA-approved, protective antigen (PA)-based vaccine (AVA, Biothrax^TM^, Bioport Corp., Lansing, MI) is limited, since a multiple-dose schedule is required for a protective-antibody titer to develop [3], [4]. Indeed, when immunization is initiated after the onset of symptoms, mortality rates due to anthrax are high [5], emphasizing the need for complimentary therapies for post-exposure events.

Antibiotic therapy is effective for the treatment of anthrax when administered soon after infection and before the onset of symptoms. Yet, due to the possible long-term survival of anthrax spores in the lungs, the updated CDC recommendations following potential exposure to aerosolized *B. anthracis* spores include a prolonged antibiotic treatment period (at least 60 days) combined with active PA-immunization [6]. However, antibiotic prophylaxis can be problematic in situations where their use is contraindicated, or in cases involving antibiotic resistant *B. anthracis* strains [7]. Furthermore, in cases where disease has progressed and a substantial amount of anthrax toxins have been delivered to the bloodstream, antibiotic treatment is of less value. In these scenarios, the passive transfer of neutralizing antibodies directed against either PA or lethal factor (LF) can provide immediate, specific and low-toxicity protection.

Neutralizing antibody titers are mainly defined by their *in vitro* activity, which is based upon the inhibition of PA-LF toxin complex-mediated cell-death (LeTx assay). It has previously been shown that the results of the LeTx assay can serve as a surrogate marker to predict an antibody's neutralization potential against anthrax challenges *in vivo* [8]. The typical method for the elucidation of PA-neutralizing monoclonal antibodies includes a wide screen and the selection of antibodies that bind to adsorbed PA (direct ELISA format), and only then the best binders were screened for their ability to neutralize PA in the LeTx assay. Although this strategy has led to the isolation of protective antibodies, it is not very effective, with each screen yielding only 3–10 candidate antibodies [9]–[14].

Here, we based our primary screen upon the LeTx assay, in order to generate a wide panel of PA-neutralizing monoclonal antibodies. Moreover, we reasoned that the level of PA-neutralizing antibodies in mouse sera will influence the screen outcome, and therefore a specific enrichment of the subset of these antibodies was desired. The results of this study support the notion that high anti-PA titers are not always associated with PA-neutralizing antibody titers. By applying an automated functionally-based screen, 101 hybridoma cell-lines exhibiting PA-neutralizing activity were isolated. The most potent antibody (cAb 29) was chimerized and shown to be highly effective in neutralizing anthrax toxin and in protecting guinea pigs against lethal doses of anthrax spores.

## Materials and Methods

### Production and purification of PA and vaccine formulation

*B. anthracis* strain V770-NPI-R (ATCC 14185) was anaerobically grown, as described previously [15]. After 24 hours of growth, bacteria were removed by microfiltration (0.2 µm), while the PA-containing supernatant was concentrated by ultrafiltration (30K M.W. cutoff) and dialyzed against 20 mM phosphate buffer (pH = 8.0). Purification of PA and LF was carried out by Q-Sepharose chromatography, essentially as described previously [8]. Formulation of PA vaccine in alum was prepared by adsorption of the purified PA at a final concentration of 50 µg/ml to Alum hydroxide gel (0.32% w/v), as described previously [8].

### PA-specific antibody titer determination using direct and indirect-ELISA

Direct-ELISA for anti-PA antibody titration was performed in 96-well microtiter plates (Nunc, Roskilde, Denmark) using an RMP200 (TECAN, Switzerland) robotic system. In this format, PA was used as the capture antigen and alkaline phosphatase-conjugated secondary antibody (anti-human, anti-mouse or anti-guinea pig IgG, for the detection of chimeric, murine and guinea pigs antibodies, respectively) was used for detection. Plates were coated with 5 µg/ml of purified PA (50 µl/well) in NaHCO_3_ buffer (50 mM, pH 9.6) at 37°C for 4 hours and then blocked with TSTA buffer (50 mM Tris pH 7.6, 142 mM sodium chloride, 0.05% sodium azide, 0.05% Tween 20, 2% BSA) at 37°C for 2 hours. Tested samples (cell culture supernatant, sera or ascitic fluids, diluted 1∶50 in TSTA) were serially diluted by two-fold dilutions, and the plates were then incubated for 2 hours at 37°C. Plates were washed with PBS containing 0.05% Tween 20, incubated with the detecting antibody, and then developed using p-nitrophenyl phosphate (Sigma St. Louis, MO, USA) as a substrate prior to measuring absorbance at 405 nm. The endpoint was defined as the highest dilution in which absorbance was higher than two standard deviations above the negative control (normal mouse serum). Antibody titers were expressed as the reciprocal endpoint dilution. Indirect-ELISA was performed using Rabbit anti-PA as the capture antibody for PA. All tests were performed in duplicate, and both negative controls (normal mouse serum) and positive standards (PA hyperimmune mouse serum) were added to each plate.

### Animal studies

All animal experiments were performed in accordance with the Israeli law and were approved by the Ethics Committee for Animal Experiments at the Israel Institute for Biological Research. Animals were maintained at 20–22°C and a relative humidity of 50±10% on a 12-h light/dark cycle, fed with commercial rodent chow (Koffolk Inc.), and provided with tap water *ad libitum*. Treatment of animals was in accordance with regulations outlined in the USDA Animal Welfare Act and the conditions specified in the Guidance for Care and Use of Laboratory Animals (National Institute of Health, 1996). Animal studies were approved by the local ethical committee on animal experiments.

### Mouse immunization

Primary immunization was performed using a group of 8 mice by s.c. injections. For the 1^st^ injection, 50 µg PA were emulsified with complete Freund's adjuvant. Two subcutaneous (s.c.) booster injections with 50 µg PA emulsified with incomplete Freund's adjuvant were administered every 2 weeks. The mouse with the highest neutralizing antibody titer received an additional i.v. boost of 5 µg PA in PBS four days prior to spleen removal.

### Cell fusion and screening

Four days after the final boost, the spleen of the mouse with the highest neutralizing antibody titer was removed and splenocytes were fused to NS0 mouse myeloma cells using polyethylene glycol, as described previously [16], [17]. After fusion, the cells were plated in 96-well cell culture plates (Nunc), at 0.5 cell per well. Selection of hybridomas was achieved by growing the cells in HAT (hypoxantine-aminopterin-thymidine) medium (Biological Industrial, Beit Haemek, Israel), and clones were observed in about 40% of the wells. Cell clones were screened for neutralizing antibody production, as described below. Positive hybridomas were cloned at least twice by limiting dilution. Cloned hybridomas were propagated both in tissue culture and in Balb/C mice.

### Cloning and expression of the murine-human chimeric 29 antibody

Chimerization of the murine 29 antibody (mAb 29) included the amplification and cloning of the murine V_H_ and V_L_ genes, encoding the antibody variable regions, followed by murine-human chimeric antibody expression. Total RNA was isolated from the murine anti-PA hybridoma 29 cells using Trizol® LS reagent (Invitrogen) and cDNA was synthesized using oligo (dT)_15_ primer, M-MLV and AMV reverse transcriptases (Promega, Madison, WI, USA). Amplification of the heavy and the light variable genes (V_H_ and V_L_) was carried out using a panel of primers directed at the 5′ of framework 1 of each gene (essentially as described [18]), and to the constant region (C_H_1 or C_k_, respectively) at the 3′ end. The mAb anti-PA 29 antibody variable segments were amplified using the primers pairs: 5′ CAG GTY CAR CTG CAG CAG YCT GG 3′; 5′ TGC AGA GAC AGT GAC CAG AGT CCC 3′ and 5′ GAY ATC CAG ATG ACH CAR WC 3′; 5′ GGG GTA GAA GTT GTT CAA GAA GC 3′ for the V_H_ and the V_L_ genes, respectively. Following sequencing, the variable genes were re-amplified using non-degenerate primers introducing restriction sites at both ends for cloning into a pCMV-based antibody expression vector( 5′ TTA AGA GGT GTA CAG TGT CAG GTT CAA CTG CAG CAG TCT 3′; 5′ GCC CTT GGT GCT AGC TGC AGA GAC AGT GAC CAG AGT CCC 3′ and 5′ CTC TGG CTG CCC GGG GCC AAA TGT GAT ATC CAG ATG ACA CAG AC 3′; 5′ GGC AGC CAC CGT ACG TTT GAT TTC CAG CTT GGT GCC TCC 3′ for the V_H_ and the V_L_ genes, respectively). The amplified heavy and light variable genes were separately purified, digested and cloned into appropriate mammalian full-length Ig expression vectors, providing each chain with a corresponding signal-peptide and constant gene, resulting in IgG1/k murine-human chimeric antibody expression.

### Stable cell line establishment

CHO cells (ACC126) were transfected with the Ig expression vector containing both heavy and light chains of the chimeric 29 antibody (cAb 29) using FuGENE 6 reagent (Roche, Mannheim, Germany). Highly anti-PA antibody producing clones were selected and expanded based on antibody levels in the supernatant, as tested by PA-specific ELISA.

### Antibody production and purification

Mouse IgG anti-PA monoclonal antibody (mAb 29) was produced in ascitic fluid, and chimeric anti-PA monoclonal antibody (cAb 29) was produced from a recombinant CHO cell line. The murine/chimeric anti-PA antibodies were purified by affinity chromatography on HiTrap Protein G/A (GE Healthcare, Uppsala, Sweden) according to the manufacturer's instructions, and dialyzed against PBS pH 7.4.

### Binding kinetics determination using Surface Plasmon Resonance (SPR)

Binding constants of murine and chimeric 29 antibodies were evaluated by SPR using a BiacoreX instrument (Biacore Inc. Uppsala, Sweden). The assay was implemented in two different formats. In the first format (direct), approximately 1000 resonance units (RU) of purified PA (10 µg/ml dissolved in 10 mM of pH 5 acetate buffer at a flow rate of 10 µl/minute) were immobilized onto a research grade CM5 sensor chip according to the manufacturer's instructions. In the second format (indirect), two additional chips were constructed, wherein, approximately 5000RU of anti-mouse or anti-human antibodies [20 µg/ml in 10 mM pH 5 acetate buffer (Jackson IR Laboratories, PA, USA)] were immobilized. All binding experiments were performed at 25^o^C in HBS-EP buffer (Biacore) at a flow rate of 20 µl/min. A blank channel was used as an on-line reference during all injections. For affinity constant determination via the direct format, 40 µl aliquots of the monoclonal antibodies (diluted to 5–100 nM) were injected over immobilized PA. In the indirect format, affinity constants were determined following injection of PA (diluted to 100–1000 nM) over immobilized monoclonal anti-PA antibodies (300 RU). Pulses of 8M urea or 100 mM H_3_PO_4_ were used to regenerate the PA-immobilized chip or anti-mouse/anti-human chips, respectively. Sensogram data were analyzed using the BIAevaluation3.2 software package. For kinetic constant determination using the PA-immobilized chip (direct-format), a bivalent analysis model was applied, whereas, for the anti-human/anti-mouse chips (indirect-format), a 1∶1 Langmuir binding model was used.

### In vitro LeTx neutralization assay

Neutralizing antibody titers were determined essentially as described previously [8], [19] by assessing their ability to protect murine macrophage J774A.1 cells (ATCC, USA) against PA/LF toxin complex (LeTx) intoxication. Tested samples were serially diluted by two-fold dilutions in TSTA buffer containing PA (5 µg/ml) and LF (2 µg/ml), and after a one hour incubation, 10 µl of each of the reaction mixture dilutions was added to the J774A.1 cells (6–8×10^5^ cells/0.2 ml). Plates were then incubated for 5 hours at 37°C in 5% CO_2_, and cell viability was monitored by the MTT (3-[4,5-Dimethylthiazol-2-yl]-2,5- Diphenyltetrazolium bromide; Thiazolyl blue) assay (absorbance was measured at 540 nm) [20]. The endpoint was defined as the highest serum dilution exhibiting 0.025 O.D absorbance units above that of the corresponding dilution of the control normal serum. Neutralizing antibody titers were expressed as the reciprocal endpoint dilutions.

### In vivo neutralization experiments

#### Rats LeTx challenge

To evaluate the ability of the 29 (murine or chimeric) antibody to protect rats against a LeTx challenge, Male Fisher 344 rats (200–250 g, Harlan, Israel) were injected intramuscularly (i.m.) with various doses of antibodies (administration volume of 100 µl in sterile PBS). Seventeen hours later, rats were anesthetized with ketamine:xylazine (50∶2 mg/kg) and injected via the tail vein (i.v.) with LeTx, containing 20 µg PA and 10 µg LF in 500 µl saline, and monitored for survival.

#### Pharmacokinetic studies

Hartley guinea pigs (females, 200–250 g, Charles River Laboratories, Wilmington, MA) were injected i.m. with antibodies diluted in 200 µl PBS. At different time points, blood samples were drawn by s.c. puncture, and antibody concentration was determined by ELISA. Pharmacokinetic parameters were calculated using the PK solutions software (Summit research services).

#### Protection against anthrax spore challenge

Hartley guinea pigs were injected i.m. with various doses of antibodies (administration volume of 200 µl in sterile PBS), and 14 hrs later challenged by s.c. administration of 40LD_50_ anthrax Vollum spores. Animal viability was monitored for at least 14 days.

## Results and Discussion

**Figure 1:**
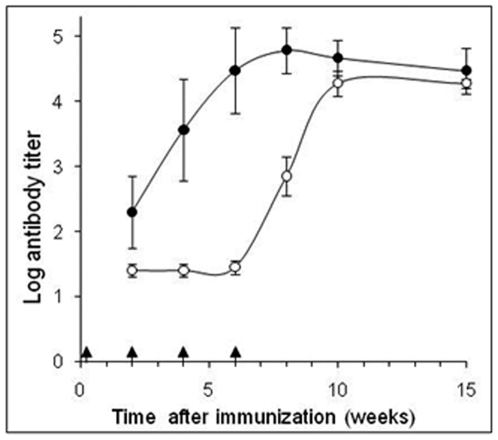
Kinetics of anti-PA and PA-neutralizing antibody development. Average titer values of total anti-PA ELISA antibodies (filled circles) of eight immunized mice and LeTx neutralizing antibody titers (empty circles) of mice 3, 4, 5 and 7, throughout the immunization process. Arrows indicate PA-immunization time points; Data points are mean±STD.

Mice were immunized with a PA-based vaccine, and titer development of either PA-neutralizing or PA-binding antibodies was carefully monitored. Two weeks after the third PA-administration (week 6), all immunized mice had developed high anti-PA titers of 30,000–60,000 (Fig. 1). Yet, at this time point, no measurable neutralizing antibody titer could be detected, and therefore another boost was given. Indeed, in four out of the eight immunized mice, neutralizing antibody titer developed gradually, reaching a maximum titer value of 30,000 by week 10. Five weeks later, the neutralizing antibody titer remained constant in these four mice (Fig. 1), yet remained negative for the other four immunized mice (data not shown). It was noticeable that, although PA-neutralizing antibodies titer increased several-fold in these mice, the titer of the total anti-PA antibodies remained unchanged. These results suggest that the continuous exposure to PA-based vaccine in these mice may have led to a specific enrichment and maturation of the desired subset of PA-neutralizing antibodies.

Fifteen weeks after the first immunization, both PA-neutralizing and PA-binding antibodies titers reached their maximum. The spleen of the mouse with the highest neutralizing antibody titer (#7) was harvested, and splenocytes were fused to NS0 mouse myeloma cells. The fusion process yielded approximately 600 hybridoma cell lines, which were screened to identify clones secreting antibodies capable of neutralizing PA activity in an *in vitro* assay. It was found that 101 clones displayed PA-neutralizing activities at different levels. The clones which demonstrated the highest neutralizing activity were chosen for further sub-cloning and characterization. At the end of this process, 19 clones with PA-neutralizing titers ranging from 100,000 to 2,300,000, were selected (Table 1).

**Table 1:** Neutralizing titers and PA-binding characteristics of selected clones.

Group	Ab #	Neutralizing titer (x10^3^)	ELISA titer (×10^3^)		direct/indirect ELISA titer ratio
			direct	indirect	
1	4	320	330	145	2.3
	19	205	410	81	5.1
	31	100	580	102	5.7
	55	820	820	410	2.0
2	10	145	13	206	0.06
	22	820	9	580	0.02
	28	205	13	145	0.09
	29	2,300	72	820	0.09
	33	290	7	820	0.01
	36	205	7	520	0.01
	38	100	36	410	0.09
	58	1,600	26	820	0.03
	68	1,600	3	1,600	0.002
3	2	360	ND^a^	820	NA^b^
	54	1,600	ND^a^	410	NA^b^
	59	410	ND^a^	1,160	NA^b^
	61	820	ND^a^	410	NA^b^
	62	820	ND^a^	820	NA^b^
	60	100	ND^a^	ND^a^	NA^b^

a ELISA titer values were below detection limits.

b Not applicable.

These 19 antibody clones were subdivided into several groups based upon a distinguishable PA-binding pattern in direct- and indirect-ELISA formats. The first group consisted of four clones, and was characterized by direct- and indirect-ELISA titers over 300,000 and 80,000 respectively (direct/indirect-ELISA ratio of≥2; Table 1). In the second group (nine clones), direct-ELISA titer was markedly lower (up to 72,000), whereas indirect-ELISA values remained similar to those of the first group, with a direct/indirect-ELISA ratio<0.1 (Table 1). While indirect-ELISA values were relatively high (>400,000) in the third group, no direct-ELISA titers could be measured for these five clones (titer<50). Notably, one clone (#60) had no measurable ELISA titer in both formats. These distinct binding patterns implied that these antibodies may recognize different sites of the target molecule, which are unequally exposed in the different ELISA formats, and work is now in progress to characterize these clones further.

It is worth noting that the isolation of 101 positive PA-neutralizing monoclonal antibody-secreting clones from a single fusion process is quite unique, and we believe it can be attributed to the fact that a neutralization-based immunization monitoring and screen were applied. These results emphasize the advantages of applying PA-neutralization functional screen, based on the following assumptions: It was shown above that 101 out of 600 clones (17%) exhibited PA-neutralizing activity to some degree, from which the top 20% were further selected. If we assume that the PA-binding characteristics of the 600 primary hybridoma clones are similar to those of the 19 selected clones, then by applying direct PA-ELISA titer as the primary limiting criterion, we would expect that only 20% (120 clones) would be positive. Consequently, we can also assume that 17% of these positive clones (20 antibodies) would be able to neutralize PA to some extent, and from that pool the top 20% (5 antibodies) would be selected for further analysis. Indeed, other studies that based their primary screens on a positive PA-binding signal in direct ELISA format were able to select only 3–10 antibodies per screen [9]–[14]. Moreover, the results presented here are in good agreement with those of other studies, in which functional screens yielded, on average, 15 clones per fusion [21].

### In vitro and in vivo LeTx neutralization

Antibody #29 (mAb 29) was chosen for chimerization and further characterization. This antibody demonstrated the highest neutralizing activity (titer of 2.3×10^6^), with ELISA titers of 72×10^3^ and 800×10^3^ in the direct and indirect formats, respectively (Table 1). A chimeric antibody, cAb 29, containing mAb 29 variable region genes (V_H_ and V_L_) fused to human constant region genes (Cγ1 and Ck, respectively) was constructed. Subsequently, the chimeric antibody was introduced into a mammalian expression-system and the recombinant antibody was stably produced by CHO cells.

**Figure 2:**
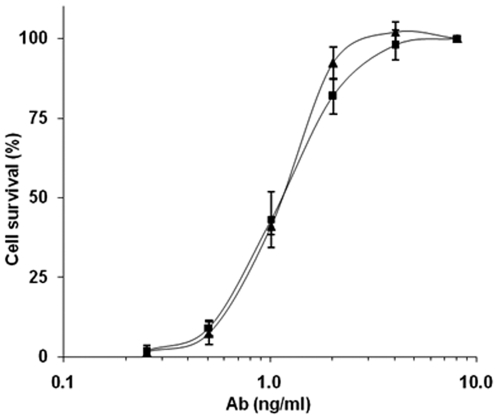
*In vitro* LeTx neutralization. Toxin complex (5 µg/ml PA and 2 µg/ml LF) was pre-incubated for one hour with increasing concentrations of cAb 29 (triangles) or mAb 29 (squares), and added to J774A.1 mouse macrophage cells for 5 hours. Cell survival was then determined by MTT, and was plotted as percent of untreated control cells. Points are mean±STD of triplicate determinants.

The ability of cAb 29 to neutralize PA-LF complex (LeTx) activity in the J774A macrophage cell line, as compared to that of the parental mAb 29, was next examined. Cells were exposed to a lethal dose of PA-LF complex in the presence of increasing antibody concentrations, and cell survival was determined 5 hrs later. Cell survival was positively correlated with both cAb 29 and mAb 29 concentrations, and full protection was achieved at 4 ng/ml for both antibodies (Fig. 2). The effective concentration of cAb 29 needed to neutralize 50% (EC_50_) of the LeTx activity was determined to be 1.3 ng/ml, comparable to that of mAb 29 (EC_50_ of 1.1 ng/ml; Fig. 2).

**Figure 3:**
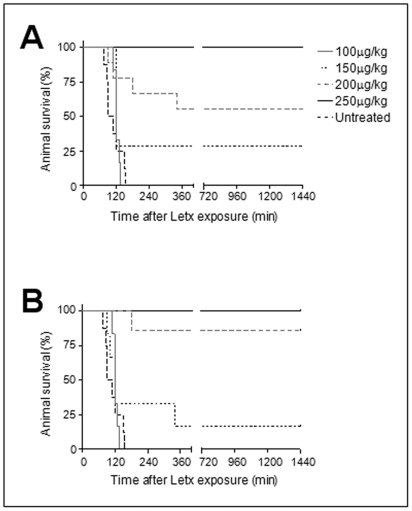
*In vivo* LeTx neutralization. Rats (n≥6 for each group) were i.m. administered with the indicated doses of (A) cAb 29 or (B) mAb 29, followed by i.v. challenge with LeTx (20 µg PA and 10 µg LF), 17 hours later. Animal survival was monitored for the next 24 hours.

To further explore the PA-neutralizing activity of cAb 29, its *in vivo* protection capacity against a LeTx challenge was tested. In this assay, antibodies were administered (i.m.) at different doses to rats, followed by an i.v. challenge with a lethal dose of LeTx 17 hrs later. All untreated control animals succumbed to this challenge, with mean time to death (MTTD) of 110 min (Fig. 3A). Treatment of animals with up to 100 µg/kg of cAb 29 did not improve survival rate, nor significantly prolong the MTTD (123 min). However, by increasing the treatment doses to 150 and 200 µg/kg, survival rates of 30 and 55% were attained (Fig. 3A). Full protection of challenged rats was achieved by a passive transfer of 250 µg/kg cAb 29. According to these results, a dose of 170 µg/kg cAb 29 was needed in order to provide 50% protection (PD_50_). The PA-neutralizing activity of mAb 29 was also evaluated under the same conditions as its chimeric counterpart, demonstrating a similar dose response (PD_50_ of 160 µg/kg; Fig. 3B). These results are in line with the *in vitro* assay results, suggesting that the chimerization process had no deleterious effect on the functional activity of the antibody.

### Binding studies

**Figure 4:**
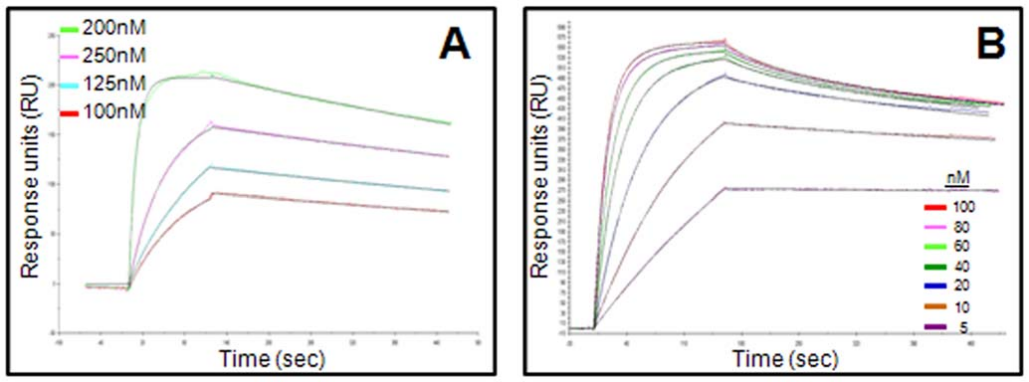
Kinetic analysis of mAb 29 and cAb 29 binding to PA. SPR sensograms obtained during injection of (A) 100–2000 nM of PA on anti-human captured cAb 29 (∼300 RU), refers to the direct format; or (B) 5–100 nM of cAb 29 on immobilized PA (∼900 RU), refers to the indirect format. Similar sensograms were obtained for the mAb 29 (results not shown).

To evaluate the PA-binding capabilities of the chimeric and murine #29 antibodies, Surface Plasmon Resonance (SPR) analysis was applied. Based on the binding characteristics, demonstrated earlier by ELISA, an indirect SPR format was applied for the kinetic measurements (Fig. 4; Table 2). Accordingly, the antibodies were attached to anti-Fc antibody pre-coated CM5 sensor chips (anti-human or anti-mouse for the cAb 29 and the mAb 29, respectively), followed by the injection of PA at different concentrations. Sensograms were fitted to a 1∶1 Langmuir model, and association (*k_on_*) and dissociation (*k_off_*) rate constants were obtained for both antibodies (Table 2). The dissociation constants (K_D_) deduced from these rate constants were similar for both antibodies, with calculated values of 6.6±2.8 nM and 8.5±4.8 nM for the chimeric and murine antibodies, respectively. When SPR analysis was performed in the direct format, in which PA was immobilized on the sensor chip, significantly lower affinities were obtained (Fig. 4; Table 2). In this assay format, a divalent analysis model was used, and K_D_ values of 37±9 nM and 44±7 nM were calculated for the chimeric and the murine antibodies, respectively (Table 2). These results are in good agreement with the ELISA binding studies, in which antibody binding to surface-immobilized PA was impaired compared to that of soluble PA (indirect format), indicating that the epitope recognized by this antibody is probably disrupted when the PA is used as the capture moiety. Taken together, the binding studies, along with the previously described *in vitro* and *in vivo* protection analyses, indicate that the chimeric form of the #29 antibody retains the binding properties of its parental murine antibody.

**Table 2:** Binding kinetics of the PA-specific antibodies.

	Immobilized PA (direct format)^a^			Immobilized anti-PA (Indirect format)^b^		
	*k*_on_	*k*_off_	*K*_D_	*k*_on_	*k*_off_	*K*_D_
	(M^−1^s^−1^)	(s^−1^)	(nM)	(M^−1^s^−1^)	(s^−1^)	(nM)
mab29	2.0×10^5^	8.9×10^−3^	44±7	0.8×10^5^	6.8×10^−4^	8.5±4.8
cAb29	2.0×10^5^	7.5×10^−3^	37±9	1.0×10^5^	6.6×10^−4^	6.6±2.8

a Binding kinetics values derived from global curve-fitting analysis using divalent analysis model.

b Binding kinetics values derived from global curve-fitting analysis using 1∶1 Langmuir binding model.

The affinity displayed here for cAb 29 (6.6 nM) is in good agreement with other PA-neutralizing antibodies described previously (10–100 nM) [22]–[25]. Moreover, cAb 29 exhibits a high association rate constant (*k*_on_ of 10^5^ M^−1^s^−1^) toward PA, and has an exceptionally slow dissociation rate constant (*k*_off_ of 6.6×10^−4^ s^−1^), a feature which could be important for long term *in vivo* protection against anthrax exposure.

### Protection against B. anthracis challenge

As shown earlier, cAb 29 is able to efficiently prevent *in vivo* intoxication with LeTx. We next examined whether this antibody can effectively protect guinea pigs against *B. anthracis* infection. To this end, the pharmacokinetic profile of the chimeric antibody and its ability to confer protection against anthrax spore exposure in a guinea pig model were determined.

### Pharmacokinetics studies in guinea pigs

**Figure 5:**
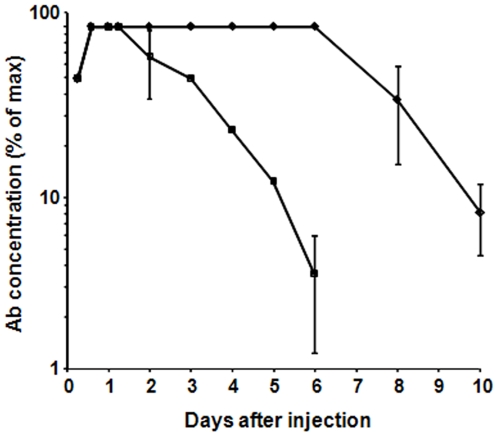
Circulatory clearance profiles of cAb 29 and mAb 29. Guinea pigs (n = 3 for each group) were i.m. administered with 5 mg/kg of either cAb 29 (diamonds) or mAb 29 (squares). Blood samples drawn at various time points were assayed for Ab concentration by ELISA. Mean±STD values are presented as percentages of maximum blood levels (C_max_).

The chimeric antibody cAb 29 and its parental murine antibody, mAb 29, were administered (i.m.) to guinea pigs, and their clearance profiles were determined by measuring the ELISA-titers of blood samples collected at different time points (Fig. 5). It was found that maximal antibody concentrations (Cmax) in the bloodstream were attained (and subsequently defined as 100% concentrations) 14 hours after injection of both antibodies. In the case of cAb 29, this level remained unchanged for the next 5 days, after which the antibody was gradually cleared from the animals' bloodstreams. The calculated mean resident time (MRT) value of cAb 29 was 6.7 days. In the case of the murine antibody, however, maximal levels were retained for only 24 hrs, displaying an MRT value of 3.3 days.

The interactions of the antibodies' constant regions with the host neonatal Fc receptor (FcRn) significantly affect the antibodies' clearance profiles. Therefore, the differences between murine and chimeric antibody pharamcokinetics are probably a reflection of their differential interactions with the guinea pig FcRn. These results are in good agreement with a previous study, in which murine antibodies cleared from guinea pigs circulation with a half-life of 3 days, while rabbit antibodies displayed considerably longer circulatory retention times (*t*_1/2_ of 9 days) [26]. It has also been shown that serum half-life prolongation of therapeutic antibodies may improve clinical outcomes, and may reduce the amount of antibodies required [27].

### Protection of guinea pigs against B. anthracis infection

**Figure 6:**
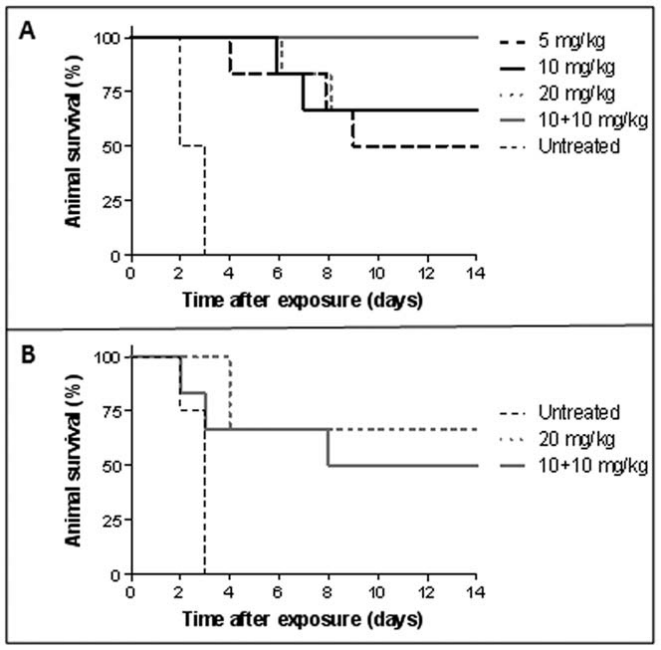
Protection of guinea pigs against anthrax infection. (A) Female Hartley guinea pigs (n = 6 for each group) were i.m. administered with the indicated doses of cAb 29, followed by s.c. challenge with 40LD_50_ of *B. anthracis* Vollum spores 14 hrs later. (B) 30 days after the first challenge, surviving animals from groups 20 and 10+10 mg/kg were re-challenged with the same dose (40LD_50_), and survival was then monitored for another 14 days.

To test the ability of cAb 29 to protect the animals against the anthrax infection, antibody doses of either 5, 10 or 20 mg/kg were i.m. administered 14 hrs prior to challenge with 40LD_50_ of *B. anthracis* Vollum spores. Under these challenge conditions, control mice (no treatment) died within 3 days post infection, with MTTD of 2.5 days (Fig. 6A; Table 3). When treated with antibody at a dose of 5 mg/kg, 50% of the animals survived the challenge with a delayed MTTD of 7 days (Fig. 6A; Table 3), significantly longer (P<0.005) than the respective control group value. Increasing the antibody dose to 10 and 20 mg/kg resulted in a minor improvement in animal survival (Fig. 6A; Table 3).

**Table 3:** Protection of Guinea pigs from anthrax infection by antibody administration.

	1^st^ challenge^a^		2^nd^ challenge^b^	
Ab dose Pre-treatment (mg/kg)	survivors/total	MTTD (days)	survivors/total	MTTD (days)
–	0/6	2.5	0/6	2
5	3/6	7		
10	4/6	6.5		
20	4/6	6.5	2/3^c^	4.3
10+10	6/6		3/6	4

a Animals were challenged with s.c administration of 40LD_50_ of *B. anthracis* Vollum spores.

b Animals that survived the 1^st^ challenge were re-infected with 40LD_50_ of *B. anthracis* Vollum spores.

C One animal was omitted from re-challenged, due to bites injury.

It was previously shown that in addition to toxin neutralization, the administration of anti-PA antibodies can markedly attenuate the dissemination of the bacteria in the early stages of infection, either by a direct anti-spore activity, or by enhancing the phagocytic uptake of bacteria by the immune system [28]–[30]. In this study, MTTD was significantly delayed even at low antibody doses, suggesting that the anti-PA antibodies affected the initial bacteria growth. Yet, at a certain time point, antibody levels were reduced and bacterial growth occurred. It was also demonstrated that soluble and membrane-bound antigens can greatly enhance antibody clearance *via* antigen-mediated elimination mechanisms [27], [31], prompting the speculation that in the current study, prolongation of anti-PA antibody residence in the bloodstream may confer improved protection. Relatively high levels of antibodies in the blood can be reached either by administering a higher antibody dose, or by applying a multiple dosing protocol [21], [32].

We therefore decided to apply two successive antibody doses of 10 mg/kg, one before the challenge and the second at day 4, based upon the pharmacokinetic profile of cAb 29 (Fig. 5). As can be seen in Fig. 6A, this protocol indeed conferred protection, and 100% of the animals survived the challenge. Since antibody pharmacokinetics may vary between animal species, a half-life of up to 3 weeks is anticipated in humans for an IgG1-type chimeric antibody. It is therefore possible that while double administration of antibody is needed for full protection in the guinea pig model, in humans, a single administration will provide equivalent and long lasting protection.

### Endogenous protective immunity against B. anthracis re-challenge

It was shown previously that following passive immunization, animals that survived anthrax challenge developed an endogenous immune response toward PA [26]. It was therefore of interest to examine whether, in our animal/antibody model, endogenous immune response was developed and whether it would confer protection against anthrax re-challenge. To this end, blood samples were drawn at day 30 after infection from animals in the groups that received cAb 29 at a total dose of 20 mg/kg (either by a single or double administration), and assessed for their ability to neutralize LeTx during the in vitro assay. Several days later, animals were re-challenged by s.c. infection with 40LD_50_ of B. anthracis Vollum spores and monitored for survival during the following 14 days. In both groups, a considerable fraction of the animals survived the second challenge, with 67% and 50% survival for the single and double administration groups, respectively (Fig. 6B; Table 3). All animals that survived the re-challenge exhibited neutralizing titer value above 100 at the time of infection, while the neutralizing titer of the non-survivors (all but one) was below 50. Anti-human antibody ELISA of these serum samples confirmed the absence of cAb 29, while all were found positive for guinea pig anti-PA titer, indicating that the neutralizing activity was endogenously derived. These results are in good agreement with previous reports, in which it was shown that the presence of PA-neutralizing polyclonal antibodies (either generated by active primary immunization or passively transferred) in sera can reliably predict protection against anthrax spore challenge [8], [26], [33]. In these studies, a neutralizing titer of 80 conferred 50% protection of guinea pigs and rabbits, and full protection was achieved at a titer of 220 and above. The ability of passive immunization (either by poly- or monoclonal antibodies) to confer protection against anthrax infection while enabling the development of endogenous immunity has been demonstrated previously in several animal models [9], [26], [33]. These results were explained by a certain degree of bacteria growth during the passive immunization process, which allowed the development of an active immune response. Moreover, it was hypothesized recently that the formation of monoclonal antibody-PA complexes may act as an immunostimulator, thereby enhancing the endogenous immune response [9].

The results of the present study strongly support the notion that immunization monitoring and primary selection of anti-PA monoclonal antibodies on the basis of their *in vitro* toxin neutralization activity can provide a large panel of antibodies with the potential to protect animals from anthrax infection. Antibody 29, which demonstrated the most potent activity among the large panel of neutralizing antibodies identified, was able to confer full protection of guinea pigs against *B. anthracis* spore infection. The data presented in this study suggest that cAb 29 is an excellent candidate for therapeutic preparations aimed at providing efficient and immediate anthrax toxin neutralization, and further evaluation of its value in other animal models is now in progress. Moreover, other neutralizing antibodies elicited in this screen and targeting different PA-epitopes are being evaluated, and will be combined in therapeutic preparations for potential synergistic effects.
